# Parents Seeking Health-Related Information on the Internet: Cross-Sectional Study

**DOI:** 10.2196/jmir.2752

**Published:** 2013-09-18

**Authors:** Aida Bianco, Rossella Zucco, Carmelo Giuseppe A Nobile, Claudia Pileggi, Maria Pavia

**Affiliations:** ^1^Medical SchoolDepartment of Health SciencesUniversity of Catanzaro “Magna Græcia”CatanzaroItaly

**Keywords:** adult, consumer health information, cross-sectional studies, health survey, Internet, Italy, questionnaires

## Abstract

**Background:**

The Internet represents an increasingly common source of health-related information, and it has facilitated a wide range of interactions between people and the health care delivery system.

**Objective:**

To establish the extent of Internet access and use to gather information about health topics and the potential implications to health care among the adult population in Calabria region, Italy.

**Methods:**

This cross-sectional study was conducted from April to June 2012. The sample consisted of 1544 adults aged ≥18 years selected among parents of public school students in the geographic area of Catanzaro in southern Italy. A 2-stage sample design was planned. A letter summarizing the purpose of the study, an informed consent form, and a questionnaire were given to selected student to deliver to their parents. The final survey was formulated in 5 sections: (1) sociodemographic characteristics, (2) information about chronic diseases and main sources of health care information, (3) information about Internet use, (4) data about the effects of using the Internet to search for health information, and (5) knowledge and use of social networks.

**Results:**

A total of 1039 parents completed the questionnaire, with a response rate equivalent to 67.29%. Regarding health-related information types, 84.7% of respondents used the Internet to search for their own medical conditions or those of family members or relatives, 40.7% of parents reported looking for diet, body weight, or physical activity information, 29.6% searched for vaccines, 28.5% for screening programs, and 16.5% for smoking cessation tools and products. The results of the multiple logistic regression analysis showed that parents who looked for health-related information on the Internet were more likely to be female (OR 1.53, 95% CI 1.05-2.25), with a high school diploma (OR 1.69, 95% CI 1.02-2.81) or college degree (OR 2.14, 95% CI 1.21-3.78), younger aged (OR 0.96, 95% CI 0.94-0.99), with chronic conditions (OR 1.94, 95% CI 1.17-3.19), not satisfied with their general practitioner’s health-related information (OR 0.6, 95% CI 0.38-0.97), but satisfied with information from scientific journals (OR 1.99, 95% CI 1.33-2.98).

**Conclusions:**

Our analyses provide important insights into Internet use and health information–seeking behaviors of the Italian population and contribute to the evidence base for health communication planning. Health and public health professionals should educate the public about acquiring health information online and how to critically appraise it, and provide tools to navigate to the highest-quality information. The challenge to public health practice is to facilitate the health-promoting use of the Web among consumers in conjunction with their health care providers.

## Introduction

The increasing use of the Internet over the past few years has allowed for a rapid and worldwide circulation and sharing of different information. The Internet also represents an increasingly common source of health-related information. An estimated 27.5% of the US adult population looked online for information about a health or medical issue in 2000 [1]. This figure increased to 40% in 2002 and to 61% in 2008 [2]. In contrast with the general information available from traditional information sources, such as magazines and television, people can systematically retrieve and obtain targeted health information through the Internet. The Internet has facilitated a wide range of interactions between people and the health care delivery system, and has become an indispensable source for the public, patients, and health care professionals to obtain information about health, diseases, and medical treatment. Health information on the Internet may make people better informed, leading to better health outcomes and more appropriate use of health service resources, although questions remain about its limitations, concerns regarding misinformation, and potential difficulties with the confidentiality of personal information. Health information on the Internet may be misinterpreted, compromising health behaviors and health outcomes.

The Internet has also grown in popularity among Italian citizens. The percentage of the Italian population between the ages of 15 to ≥75 years that uses the Internet has increased from 32.3% in 2005 to 52.1% in 2012 [3]. Understanding the extent to which the Internet is being used to obtain health-related information and the effects it has on health care use would help identify the benefits that are being realized and provide a context for fruitful discussions of the current and future role of the Internet in health care. Some advanced countries, such as the United States, have accumulated research on health information-seeking behavior through a number of population-based surveys [2,4,5], whereas studies investigating the active health information-seeking behavior of the Italian public are scanty.

Therefore, this study was designed to establish the extent of Internet access and use to gather information about health topics and the potential implications in health care among the adult population in the Calabria region, Italy.

## Methods

### Overview

This cross-sectional study was conducted from April to June 2012. The sample comprised 1544 adults aged ≥18 years selected among parents of public school students in the geographic area of Catanzaro in southern Italy.

A 2-stage sample design was planned. We divided the target population into kindergarten, elementary, middle, and high schools, which were used as first-stage sampling units. A simple randomization technique with replacement was adopted in selecting each school. A sampling frame of all students was then assembled for each selected school. At the second stage of sampling, a sample of students was randomly selected from each school. During school hours, each selected student was given a letter summarizing the purpose of the study and pointing out the voluntary and confidential nature of participation, an informed consent form, and a questionnaire to deliver to their parents.

Before starting data collection, a meeting with the head of each selected school was arranged to present the project and to discuss the research strategy.

The sample size was determined before study initiation. It was calculated assuming that 50% of the respondents look online to obtain health-related information in accordance with prior European studies, a margin of error of 5%, and a 95% confidence level. Consequently, a sample of 385 parents was sought. Anticipating a response rate of 45%, a total sample size of 789 parents was needed. We included an additional 250 parents in case the response rate among Internet users was not adequate.

The questionnaire was developed based on previous studies [6-8] and was pretested for length and content on a sample of 47 potential respondents.

The final survey was 2 pages in length, designed to be completed within 10 minutes and formulated into 5 sections: (1) sociodemographic characteristics (gender, age, marital status, level of education, and employment); (2) information about chronic diseases and the main sources of health care information; (3) information about Internet use, including whether participants had a computer at home and/or access to the Internet and, if the parent had access to the Internet, if he or she used the Web to search for health information; (4) data about the effects of using the Internet to search for health information; and (5) knowledge and use of social networks.

Each section elicited responses in a variety of formats: closed-ended questions with multiple answers possible, yes or no questions, and open-option questions. The questionnaire culminated with the option of providing additional comments.

The study protocol was approved by the Ethics Committee of the Mater Domini Hospital of Catanzaro (Italy) (2012/04/20).

### Statistical Analysis

Multivariable backward stepwise logistic regression models were constructed to determine the explanatory variables independently related to dichotomous measures of whether or not the Internet was used for health-related information seeking. The model building strategy included the following steps: (1) univariate analysis of each variable considered, using the appropriate test statistic (chi-square test or *t* test); (2) inclusion of any variable whose univariate test has a *P* value <.25; (3) the results of the logistic regression analysis are presented as odds ratios (ORs ) and 95% confidence intervals (CIs ). A 2-sided *P* value for all tests of <.05 was considered a statistically significant difference. The significance level for a variable’s entry to the model was set at .2 and at .4 for removal.

The following explanatory variables were included in the model: gender (male=0, female=1), age (continuous), satisfaction about information received from general practitioner (GP; dissatisfied=0, satisfied=1), education level (3 categories: elementary/middle school=1, high school=2, university degree=3), number of visits to the GP (ordinal, once per year or less=1, 3-4 times per year=2, once per month=3, 2-3+ times per month=4) and presence of chronic conditions (no=0, yes=1), satisfaction about information received from TV/radio (dissatisfied=0, satisfied=1), and satisfaction about information received from scientific journals (dissatisfied=0, satisfied=1).

Stata version 11 (StataCorp LP, College Station, TX, USA) statistical software package was used in conducting all data analyses.

## Results

Ten public schools were selected. A total of 1039 parents completed the questionnaire, with a response rate equivalent to 67.29% (1039/1544). The main sociodemographic characteristics of the study population are shown in Table 1. The GP represented the main source of health-related information among 65.16% (677/1039) of respondents. Other sources of health information were Internet (44.46%, 462/1039), TV/radio (27.62%, 287/1039), and scientific journals (15.21%, 158/1039).

The respondents’ Internet use pattern is reported in Table 2. Most respondents (95.67%, 994/1039) had a personal computer at home and 58.16% (602/1035) used one at work. Among computer users, Web browsing was very frequent; most (85.76%, 891/1039) used the Internet, and almost three-quarters (72.6%, 647/891) had been using the Internet for 4 years or longer. Of the Internet users, 83.2% (741/891) reported Internet access at home, and 83.1% (740/891) used the Internet to search for health-related information. Regarding health-related information types, 84.7% (627/740) of respondents used the Internet to search for their own medical conditions or those of family members or relatives. When data about lifestyle and preventive care utilization were explored, 40.7% (301/740) of parents reported looking for diet, body weight, or physical activity information, 29.6% (219/740) for vaccines, 28.5% (211/740) for screening programs, and 16.5% (122/740) for smoking cessation tools and products. In all, 60.4% (447/740) of users searched for information about national and/or local health services providers. Most users (96.6%, 715/740) had never bought drugs or vitamins online. Only 22.9% (170/740) of parents communicated via email with their GP or specialists, although 61.8% (352/570) would like to do so.

Among parents who used the Internet to search for health-related information, 81.2% (599/738) said that it improved their understanding of health care issues and they learned more about an illness or a specific symptom, and 23.0% (170/738) reported that they used the Internet to obtain more information than that provided by their GP. More than half (58.5%, 432/738) considered the retrieved information very useful and 49.1% (362/738) stated that they were able to find online answers to their health care questions (data not shown).

Data quality on the Web may be of concern and criteria used by respondents were investigated. In all, 59.9% (438/731) stated that they visited websites sponsored by physicians or medical associations, and 16.9% (125/740) did not care about health-related information reliability. At univariate analysis, the Internet use for searching health-related information was significantly higher among female (χ^2^_1_=6.0, *P*=.01), younger participants (*t*_889_=3.6, *P*<.001), with a higher level of education (χ^2^_1_ for trend=14.1, *P*<.001), who were more frequently unsatisfied by GP health-related information (χ^2^_1_=9.7, *P*=.002), but were satisfied about information received by scientific journals (χ^2^_1_=22.0, *P*<.001) or TV/radio (χ^2^_1_=4.6, *P*=.03; see Table 3). It was also higher for those who reported visiting their GP less than 5 times in a year but this did not meet statistical significance (χ^2^_1_ for trend =2.8, *P*=.09). The results of the multiple logistic regression analysis substantially confirmed the findings of the univariate analysis. Indeed, parents who looked for health-related information on the Internet were more likely female (OR 1.53, 95% CI 1.05-2.25), with a high school diploma (OR 1.69, 95% CI 1.02-2.81) or college degree (OR 2.14, 95% CI 1.21-3.78), younger (OR 0.96, 95% CI 0.94-0.99), with chronic conditions (OR 1.94, 95% CI 1.17-3.19), not satisfied with their GP’s health-related information (OR 0.6, 95% CI 0.38-0.97), but satisfied about information received by scientific journals (OR 1.99, 95% CI 1.33-2.98; see Table 3).

Regarding influence of information obtained from the Web on health care–related behavior, 69.2% (512/740) of the Internet users indicated that the information they found modified the way they thought about their health. In particular, 57.8% (296/512) reported they had become more interested in health issues, and 36.7% (188/512) were less confused about health problems. Moreover, 43.5% (322/740) of the eligible parents started paying more attention to eating habits and food, and 33.9% (169/498) and 18.7% (138/738) started or increased physical activity and increased participation in screening programs, respectively (data not shown).

Among participants who used the Internet to search for health-related information, only 25.4% (188/740) discussed this with their GP. A total of 78.5% (581/740) of the eligible respondents believed that Internet use had not changed their relationship with their GP in any way; 13.4% (99/740) believed it had a positive effect and 8.1% (60/740) believed it had a negative effect. After using the Internet, 12.7% (94/740) of the sample had reduced their frequency of GP visits.

Regarding social networks, more than half of parents (56.9%, 505/886) said they had a profile on a social network; the most used social platform was Facebook (97.6%, 493/505). Almost half of these parents accessed it daily (49.5%, 250/505). A total of 40.8% (206/505) of participants used Internet for health-related social support and access to open forums or groups focused on medical issues in particular to ask for help in the management of a disease or a symptom (60.2%, 124/206) or to share illness experiences (43.7%, 90/206).

**Table 1:** Selected characteristics of the study population.

Characteristic		Overall sample (N=1039)^a^	Internet users (n=891)^a^
**Gender, n (%)**			
	Female	704 (67.76)	590 (66.22)
	Male	335 (32.24)	301 (33.78)
**Age group (years), n (%)**			
	18-35	223 (21.46)	215 (24.13)
	36-40	240 (23.09)	213 (23.91)
	41-45	285 (27.43)	252 (28.28)
	46-50	191 (18.39)	144 (16.16)
	>50	100 (9.63)	67 (7.52)
Age, mean (SD)		41.2 (7.4)	40.4 (7.3)
**Marital status, n (%)**			
	Married	865 (83.25)	733 (82.27)
	Single/divorced/separated/widowed	174 (16.75)	158 (17.73)
**Education level, n (%)**			
	No formal education/completed elementary/middle school	195 (18.76)	106 (11.89)
	Completed high school	518 (49.86)	465 (52.18)
	Holds a bachelor’s degree or any college degree	326 (31.38)	320 (35.93)
**Employment status, n (%)**			
	Unemployed/housewife/retired	312 (30.44)	215 (24.51)
	Employed	520 (50.73)	476 (54.28)
	Professional/autonomous work	193 (18.83)	186 (21.21)
Chronic conditions, n (%)		243 (23.38)	202 (22.67)
**Frequency of visits to general practitioner**			
	Once per year or less	279 (26.85)	259 (29.07)
	3-4 times per year	342 (32.91)	308 (34.57)
	Once per month	276 (26.57)	226 (25.35)
	2-3 times per month	142 (13.67)	98 (11.01)
**Sources of health-related information,** ^b^ **n (%)**			
	General practitioner	677 (65.16)	558 (62.62)
	Internet	462 (44.46)	461 (51.73)
	TV/radio	287 (27.62)	228 (25.59)
	Scientific journals	158 (15.21)	150 (16.83)
	Magazines/books	43 (4.14)	35 (3.92)
	Family members/friends/colleagues	26 (2.50)	24 (2.69)

^a^Total may not always sum to N because of missing data.

^b^Multiple responses allowed.

**Table 2:** Personal computer and Internet use patterns of respondents.

Use of Internet (number of respondents)		n	%
Having a personal computer at home (1039)		994	95.67
Having a personal computer at the workplace (1039)		602	58.16
Use of Internet (1039)		891	85.76
**Duration of Internet use (891)**			
	<1 year	51	5.72
	1-3 years	193	21.66
	4-6 years	264	29.62
	7-10 years	188	21.09
	>10 years	195	21.91
Internet use to search for health-related information (891)		740	83.05
**Webpages visited for health-related information** ^a^ **(731)**			
	Physicians or medical association	438	59.91
	Ministry of Health	206	28.18
	Chat	186	25.44
	Hospitals	85	11.62
	National scientific societies	81	11.08
	International organizations competent for health	69	9.43
	International scientific societies	48	6.56
	Local organizations competent for health	30	4.10
	Other	16	2.18
Internet use to better understand the meaning of a medical term (740)		672	90.81
**Internet use to search more information about each of the following** ^a^ **(740)**			
	Disease diagnosis	636	85.94
	Own, family member, or relative health status	627	84.73
	Disease treatment	521	70.40
	Health services provider	447	60.40
	Drugs	393	53.11
	Diet, weight, or physical activity	301	40.67
	Vaccines and/or vaccinations	219	29.59
	Screening programs	211	28.51
	Smoking cessation	122	16.48
	Internet use to buy drugs or vitamins (740)	25	3.37
	Use email to communicate with the general practitioner (740)	170	22.97
	Talk with general practitioner about information retrieved on the Internet (740)	188	25.41
	Creating an online profile (886)	505	56.99
**Social networks visited** ^a^ **(505)**			
	Facebook	493	97.62
	Twitter	62	12.27
	Google+	47	9.31
	LinkedIn	14	2.72
	MySpace	9	1.78
	Viadeo	2	0.39
**Frequency of visiting online social networking sites (505)**			
	Never/almost never	38	7.52
	3-4 or less times per month	43	8.51
	1 or less times per week	60	11.88
	2-4 times per week	69	13.66
	5-6 times per week	45	8.91
	Daily	250	49.52
Internet use for health- related social support (505)		206	40.79

^a^Multiple responses allowed.

**Table 3:** Univariate and multivariate analyses of Internet use for health-related information seeking according to various explanatory variables.

Variable		Univariate						Multivariate	
		Mean (SD)	*t*_889_	*P*	Health-related information seekers (n=740)^a^			OR	95% CI
					n (%)	χ^2^_1_ or χ^2^_1_ for trend	*P*		
**Gender**						6.0	.01		
	Male				237 (78.7)			1.00^b^	
	Female				503 (85.2)			1.53	1.05-2.25
**Education level**						14.1	<.001		
	Elementary/middle school				74 (69.8)			1.00^b^	
	High school				387 (83.2)			1.69	1.02-2.81
	College degree				279 (87.2)			2.14	1.21-3.78
**Age (years)**			3.6	<.001					
	Users	40.05 (7.35)						0.96	0.94-0.99
	Not users	42.37 (6.83)							
**Chronic conditions**						2.4	.12		
	No				565 (82)			1.00^b^	
	Yes				175 (86.6)			1.94	1.17-3.19
**Frequency of visits to general practitioner**						2.8	.09		
	Once per year or less				219 (84.6)			—^c^	—^c^
	3-4 times per year				266 (86.4)				
	Once per month				174 (77)				
	2-3 times per month				81 (82.6)				
**Satisfaction with information received from general practitioner**						9.7	.002		
	Dissatisfied				225 (89.3)			1.00^b^	0.38-0.97
	Satisfied				515 (80.6)			0.6	
**Satisfaction with information received from scientific journals**						22.0	<.001		
	Dissatisfied				381 (77.8)			1.00^b^	
	Satisfied				349 (89.7)			1.99	1.33-2.98
**Satisfaction with information received from TV/radio**						4.6	.03		
	Dissatisfied				361 (80.4)			—^c^	—^c^
	Satisfied				374 (85.8)				

^a^Total may not always sum to N because of missing data.

^b^Reference category.

^c^Removed by the model.

## Discussion

### Principal Findings

The Internet is broadly recognized as a potentially important instrument for transforming medical care and public health [5,9]. It offers tremendous promise as a health communication and education tool [10,11], and it could be a key resource in health behavior change interventions and programs [12]. This study provides an outline of the prevalence of Internet use among adults aged 18 years and older, describes the variables associated with its use related to health or medical issues, and the impact of the information on health-related behaviors.

Internet access is a widely diffused technology in the surveyed area; approximately 85% of our sample accessed it. Searching for health information online appeared to be a prevalent activity among the population, and the Internet is considered the second most important source of health-related information, preceded only by health professionals who are still the main source of health information by far. Nevertheless, among Internet users, 83.1% reported that they look online to obtain health-related information for themselves, family members, or relatives and the prevalence was higher than those reported in the United States in 2009 (61%) [2], in Japan in 2007 (24%) [13], and in other European countries in 2005 (42%) and in 2007 (52%) [14]. However, comparisons with previous studies must be interpreted cautiously because the time frame considered (in the present survey “at least once”) may influence the prevalence of Internet use for health-related information. Indeed, the outcome is much less prevalent if measured in a shorter time frame; in the studies in which respondents had a narrower time frame prevalence diminished substantially.

The findings from this investigation shed considerable light on the variables related to Internet use for health-related information. Multivariate data analysis results regarding health information seekers characteristics were consistent with many preceding studies pointing out that younger people, females, those with a higher level of education, not satisfied with their GP’s health-related information, and those with chronic conditions reported more frequent access to the Internet to seek health-related information [15-18]. In general, education was reported as 1 of the strongest predictors of whether someone has access to the Internet [2]. We tested the hypothesis that more educated participants were more likely to engage in a search for health-related information, and the current research found that only 10% of Internet users with less than a high school degree do so compared to 90% of participants with a high school or college degree. Moreover, we found that who looked for health-related information on the Internet was more frequently affected by chronic diseases and those not satisfied with the information provided by their GP. Because of health consultation time constraints, patients are often left with a sense of frustration and dissatisfaction with the information provided, whereas they would like to be fully informed and be part of the medical decision-making. Patients with chronic conditions usually use the Internet to gain supplemental knowledge to that received from their physician. Moreover, those patients’ access to support groups, typically targeted to a particular disease, allow the ill individual to gain coping mechanisms.

As reported in previous research [18], in this study many adults surfed the Internet for additional information about disease diagnosis (85.9%) and/or treatment (70.4%). This result is important, particularly because only 25.4% of them talked with their GP about the information retrieved from the Internet. The behavioral discrepancy between searching for information on the Internet and not using this information with health professionals might be because of user conflict derived from not trusting health professionals whose attitude and behavior are incompatible with the information from the Internet. We supposed that, in response to the Internet-informed patient, the patient-health professional relationship can become health professional-centered with the health professional exerting his or her expert opinion. They will use the short consultation time to quickly and authoritatively steer the patient toward their choice of action. This figure could be an issue because the scientific quality of information is difficult to evaluate by the public. Although only limited evidence shows that Internet use for health-related information results in harmful health outcomes [19], past research suggests that many adults surf health information online to self-diagnose, to seek information on alternative treatments or medicine, or to engage in health care strategies inconsistent with medical recommendations [18]. In our opinion, it would be appropriate to use the Internet as a supplement to health services rather than as a replacement, and to share the information with one’s GP. However, health professionals should be mindful of patients’ desire for health information [20]. The triangulation of patient-Web-practitioner may have remarkable potential for improving the physician-patient relationship to include enhanced communication, shared decision-making, and more efficient use of clinical time.

In the present survey, we also examined the prevalence of Internet use related to wellness information (ranging from 40.7% for diet, weight, or physical activity to 16.5% for smoking cessation). Most health risks in the modern world are related to lifestyles (eg, overweight, unhealthy diet, physical inactivity, and smoking), and the observation that individuals actively sought these information could be a key in the prevention and management of risk conditions. This suggests that the Internet could provide an efficient channel for primary health promotion and disease prevention activities, encouraging many individuals to search the Internet for health information to maintain a healthy lifestyle. Results of the present survey may be relevant for future development and implementation of Web-based interventions aimed at improving lifestyle behavior. An Internet-based lifestyle intervention may overcome significant barriers to preventive counseling and facilitate the incorporation of evidence-based lifestyle interventions into primary care [21], providing methods and motivation for behavior change. It may create awareness of unhealthy behavior before chronic disease symptoms are present, by providing information about healthy behavior. This is a crucial step for those not yet ready for behavioral change [18].

In our Internet users sample, 22.9% reported using email to communicate with their GP, and this finding suggests that online communication with GPs is not widespread in Italy. Communication via email between health providers and health consumers represents an important topic that should be addressed in the future because it may offer opportunities for the public to interact interpersonally with health professionals [22].

### Limitations

Although the findings of this study are meant to stimulate discussion about the role of the Internet in health promotion and disease prevention, there are several limitations to acknowledge. First of all, it should be noted that, because this study has a cross-sectional design, the relationship between the predictor variables and the dependent variables should not be taken as a cause-and-effect relationship; the study is able only to describe general associations. Second, although the data were produced using a rigorous methodology, they are from self-report assessments and may reflect certain biases as a result. As with any survey based on a self-administered questionnaire, information may not be entirely accurate, primarily because of the long time frame used in the study that may introduce recall bias. On the other hand, longer time frames are useful for formulating broad prevalence estimates in a context in which no data are yet available.

Third, we collected data in 1 Italian region, which might not represent all adult population in Italy; therefore, concern about generalizability and comparability of the findings may arise. However, we are confident that the findings of the study may be representative of the Southern regions and may be referred to the whole country. Moreover, it is well known that the ability to generalize from a sample is limited by the sample frame, and we selected participants from parents of children and students attending kindergarten, elementary, middle, and high schools. This population, compared to the general population, probably underestimates people older than 50 years and excludes those who do not have children. However, we believe that these characteristics do not have a substantial impact on Internet use for health-related information because adults aged between 18 and 49 years are more likely than older adults to participate in social technologies related to health [2]. Therefore, our results may be generalized to adult Internet users.

### Conclusions

Despite the limitations identified, our analyses provide important insights into Internet use and health information-seeking behaviors of the Italian population and contribute to the evidence base for health communication planning. Health and public health professionals should educate patients about acquiring health information online and critically appraising it [23], and provide tools for them to navigate to the highest-quality information. Understanding health information-seeking behavior in relation to use of the Internet is timely and important, given the rapid increase in the amount of information available online and the increasing influence of online health information seeking on health behaviors, health processes, and health outcomes. The challenge to public health practice is to facilitate the health-promoting use of the Web among consumers in conjunction with their health care providers.

## Abbreviations

GP: general practitioner
